# CHEK2 is a potential prognostic biomarker associated with immune infiltration in clear cell renal cell carcinoma

**DOI:** 10.1038/s41598-023-49316-6

**Published:** 2023-12-11

**Authors:** Qihang Wu, Cheng Fang, Xue Wang, Shuaishuai Huang, Guobin Weng

**Affiliations:** 1 Health Science Center, Ningbo University, Ningbo, Zhejiang China; 2 Department of Urology, Ningbo Yinzhou No. 2 Hospital, Ningbo, Zhejiang China; 3 Urology and Nephrology Institute of Ningbo University, Ningbo Yinzhou No. 2 Hospital, Ningbo, Zhejiang China

**Keywords:** Computational biology and bioinformatics, Biomarkers

## Abstract

Checkpoint kinase 2 (CHEK2) plays a crucial role in responding to DNA damage and is linked to diverse cancer types. However, its significance in the prediction of prognosis and impacts on the immune status of clear cell renal cell carcinoma (ccRCC) remains unclear. This study aimed to identify the role of CHEK2 in prognosis and immune microenvironment of ccRCC. We analyzed transcriptome and clinicopathological data from the cancer genome atlas (TCGA) database and conducted functional enrichment analysis to explore molecular mechanisms. The relationship between CHEK2 and immune infiltration was evaluated, and drug sensitivity analysis was performed using the CellMiner database. The results showed that CHEK2 was an independent predictor of ccRCC prognosis and was closely associated with immune-related processes. Additionally, high expression of CHEK2 was linked to resistance to certain targeted drugs. These findings suggest that CHEK2 could serve as a biomarker for ccRCC, providing insights into tumor immune microenvironment alterations and immunotherapeutic response. Further investigation is needed to fully understand the potential of CHEK2 as a prognostic predictor and therapeutic target for ccRCC.

## Introduction

Renal cell carcinoma (RCC) is among the 10 most frequent cancers diagnosed worldwide^1^. Clear cell renal cell carcinoma (ccRCC) is an RCC type related to the highest morbidity and accounts for a large number of RCC-associated deaths^2^. Clinically, about a quarter of RCC cases are metastatic at first diagnosis, and 30% of patients with localized disease exhibit relapsed metastasis after curative nephrectomy^3^. Despite the wide range of available treatment methods, including surgical resection, radiation, chemotherapy, targeted therapy, and novel immunotherapeutic agents, the prognosis of patients with metastatic RCC is dismal. As a result, it is critical to explore potential biomarkers and novel anticancer candidates with enhanced selectivity and efficacy.

The DNA damage response (DDR), which has evolved in cells to prevent DNA damage, is a complicated network of biochemical pathways ensuring genomic stability. Genome instability is an important feature of cancer^4^. Due to the genetic instability of cancer cells, mutations and tumor heterogeneity are common^5^. These characteristics imply that the dysregulation of DDR-related pathways underlies the propensity for the enhanced proliferation, growth, and tumorigenesis of cancer cells^6^. Abnormalities in DDR genes have been reported in various cancers^7,8^. A recent study of 844 early‑onset renal cancers tested using a multi-gene panel identified checkpoint kinase 2 (CHEK2) as the most common pathogenic variant in DDR genes^9^. The *CHEK2* gene encodes the CHK2 protein, which is a vital factor that responds to DNA double-strand breaks (DSB)^10^. Notably, DSB is often the most destructive type of DNA damage and disrupts genomic integrity^11^. Recently, the expression patterns and functions of CHK2 in tumors have increasingly drawn interest and have thus emerged as the research hotspot in molecular oncology. High CHK2 expression is tightly associated with adverse tumor features, such as the recurrence, progression, and metastasis of many malignant tumors^12,13^. Nevertheless, the connection as well as the clinicopathological value of CHEK2 expression level in ccRCC remains unclear and deserves more studies.

The present work focused on evaluating the expression of CHEK2 and its prognostic value in ccRCC patients, as well as its correlation with immune properties in the tumor microenvironment (TME).

## Materials and methods

### Data extraction and preprocessing

The gene transcriptome profiles, mutation data, and corresponding clinical information on ccRCC cases were gathered from the cancer genome atlas (TCGA; https://portal.gdc.cancer.gov/) database, comprising 541 ccRCC and 72 non-carcinoma samples. Then, we excluded cases that lacked crucial clinical data and matched the *CHEK2* gene matrix with the corresponding clinical characteristics for further analyses.

### Analysis of *CHEK2* gene expression

The online tumor immune estimation resource (TIMER; https://cistrome.shinyapps.io/timer/)^14^, developed to investigate immune infiltrates comprehensively and systemically in different cancers, was employed to assess the expression of CHEK2 across diverse cancer types in the TCGA database. Then, the TCGA-KIRC cohort was explored to evaluate the different expression levels of CHEK2 mRNA between the ccRCC group and the control group. Moreover, CHEK2 protein levels were compared between normal kidney samples and ccRCC samples using the “CPTAC analysis” module of the UALCAN database (http://ualcan.path.uab.edu/)^15^. The available immunohistochemical images showing healthy kidney and ccRCC samples were acquired from the human protein atlas (HPA; https://www.proteinatlas.org) database^16^. Furthermore, we assessed the expression of CHEK2 based on various clinicopathological characteristics in ccRCC samples from both TCGA and UALCAN.

### Cell culture

Human ccRCC cell lines 786-O, ACHN, and healthy kidney tubular epithelial HK-2 cells were offered by the cell bank of the Chinese Academy of Sciences. Human ccRCC OS-RC-2 cells were provided by American Type Culture Collection (ATCC). ACHN and HK-2 cells were cultivated in Dulbecco’s modified eagle medium (DMEM; HyClone) including 10% fetal bovine serum (FBS; Invitrogen), whereas 786-O and OS-RC-2 cells were cultivated in Roswell Park Memorial Institute (RPMI; HyClone) 1640 medium containing 10% FBS. The incubation conditions were 37 °C under 5% CO_2_.

### Western blot analysis

To perform Western blot analysis, RIPA buffer (Solarbio) including a protease inhibitor (1% PMSF; Solarbio) was used to lyse the Cultured cells. Then, 30-µg protein lysates were separated on 10% SDS-PAGE gel and transferred onto a 0.45-µm PVDF membrane (Millipore). After the membranes were placed in TBST buffer including 5% non-fat milk to block, specific primary antibodies, including CHEK2 (rabbit polyclonal, 1:1000; 13954-1-AP, Proteintech) and β-actin (rabbit monoclonal, 1:50,000; AC026, ABclonal) were added to the membranes and incubated overnight at 4 °C. After thorough rinsing with TBST buffer, the membranes were treated with goat anti-rabbit IgG secondary antibodies (1:5000, AS014, ABclonal) at room temperature for another 1 h. Then, the blots were treated with enhanced chemiluminescence (ECL) reagent (Beyotime) for detection, and the protein bands were scanned and analyzed using a gel imaging system (Tanon, China).

### Analysis of prognosis

The comprehensive online platform gene set cancer analysis (GSCA; http://bioinfo.life.hust.edu.cn/web/GSCA/) has been prepared for the analysis of multiomics data across 33 cancer types from TCGA^17^. In the current work, we utilized GSCA to examine the associations between CHEK2 mRNA level and overall survival (OS), progression-free interval (PFI), disease-specific survival (DSS), and disease-free interval (DFI) for ccRCC patients. In addition, the ccRCC patients were divided into high- or low-expression groups based on the median values of the above parameters. Moreover, independent prognostic features of ccRCC patients were evaluated with univariate and multivariate Cox regression.

### Differentially expressed genes (DEGs) and functional enrichment

The DEGs in the high- versus low-expression groups were identified using the “limma” package, with thresholds set at an adjusted *P* value < 0.05 and | log2 (Fold Change, FC) |> 1. The 30 most upregulated or downregulated DEGs were visualized with the “pheatmap” package. Moreover, gene ontology (GO) and Kyoto Encyclopedia of Genes and Genomes (KEGG) analyses were conducted with the use of the “clusterProfiler” package.

### Immune microenvironment landscape and mutation analysis

The ESTIMATE algorithm was adopted for estimating the stromal/immune cell ratio and the “estimate” package was employed to calculate stromal/immune/ESTIMATE scores for ccRCC patients. Besides, we applied the CIBERSORT algorithm with the purpose of assessing the proportions of 22 tumor-infiltrating immune cells (TIICs). The “GSVA” package was adopted for determining the immune function scores for all cases. We then examined the relation of CHEK2 with immune checkpoints in ccRCC patients. Moreover, tumor immune dysfunction and exclusion (TIDE) scores for all ccRCC cases were examined from the TIDE database (http://tide.dfci.harvard.edu/login/) to predict the likelihood of immunotherapeutic response. Finally, we explored different somatic mutations in the high- versus low-expression groups on the basis of the “maftools” package.

### CHEK2-interacting molecules and functional enrichment

The GeneMANIA database (http://genemania.org/search/homo-sapiens) was used to create the network of gene–gene interactions related to CHEK2^18^. GO and KEGG analyses were carried out on the 20 most significant CHEK2-binding proteins.

### Drug sensitivity of CHEK2

The CellMiner database (https://discover.nci.nih.gov/cellminer/home.do) and query tool was used to obtain the RNA-seq expression profiles and NCI-60 compound activity data to evaluate the drug sensitivity of CHEK2^19^. Drugs approved by FDA were chosen for investigation.

### Statistical analysis

The differences in expression patterns and correlation between the two groups were explored using Wilcoxon rank-sum and Spearman’s rank tests, respectively. Student’s t-test was adopted for analyzing pairwise differences between the groups. Kaplan–Meier survival curves were plotted to examine the survival outcome by log-rank test. Hazard ratios (HRs) were calculated with the Cox proportional hazard regression model. Based on the statistical software GraphPad Prism version 9.0 and R version 4.1.3, statistical analyses were performed. *P* values < 0.05 suggested significant differences between the groups.

## Results

### CHEK2 expression increased in cancer tissues

**Figure 1:**
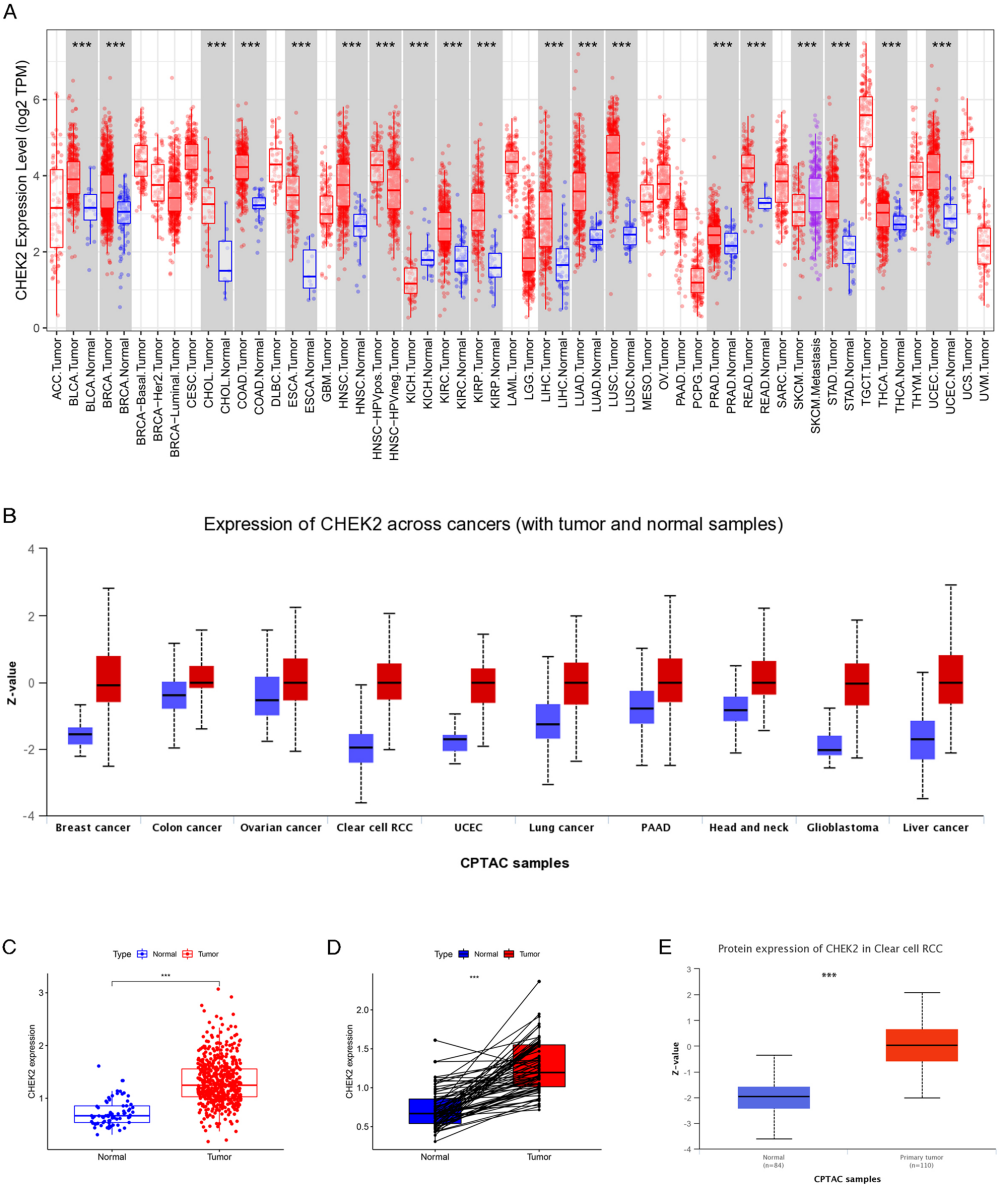
The expression of CHEK2 in different datasets. (**A**) The mRNA expression of CHEK2 in pan-cancer from TIMER database; (**B**) The protein expression profile of CHEK2 in pan-cancer from UALCAN database; (**C**) The mRNA expression levels of CHEK2 in ccRCC, normal samples and paired adjacent normal tissues (**D**) was analyzed based on TCGA-KIRC cohort; (**E**) The protein expression of CHEK2 in ccRCC from UALCAN database. *P < 0.05, **P < 0.01, ***P < 0.001.

First, the expression levels of CHEK2 in different tumor types in the TIMER database were evaluated. All tumors except for chromophobe renal cell carcinoma (KICH) showed elevated levels of CHEK2 expression compared to normal cells (Fig. 1A). Similarly, CHEK2 protein expression was upregulated in all cancer types on the basis of the data from the UALCAN database (Fig. 1B). Moreover, CHEK2 expression increased significantly compared to normal kidney tissue, according to the TCGA-KIRC cohort analysis (Fig. 1C). In paired specimens, CHEK2 levels of the ccRCC group increased substantially relative to matched normal tissue (Fig. 1D). Furthermore, CHEK2 protein levels in ccRCC samples were examined using the UALCAN database (Fig. 1E). Collectively, both mRNA and protein expression levels of CHEK2 showed obvious increase in ccRCC samples than in normal kidney tissue.

### CHEK2 levels in clinical samples and cell lines

**Figure 2:**
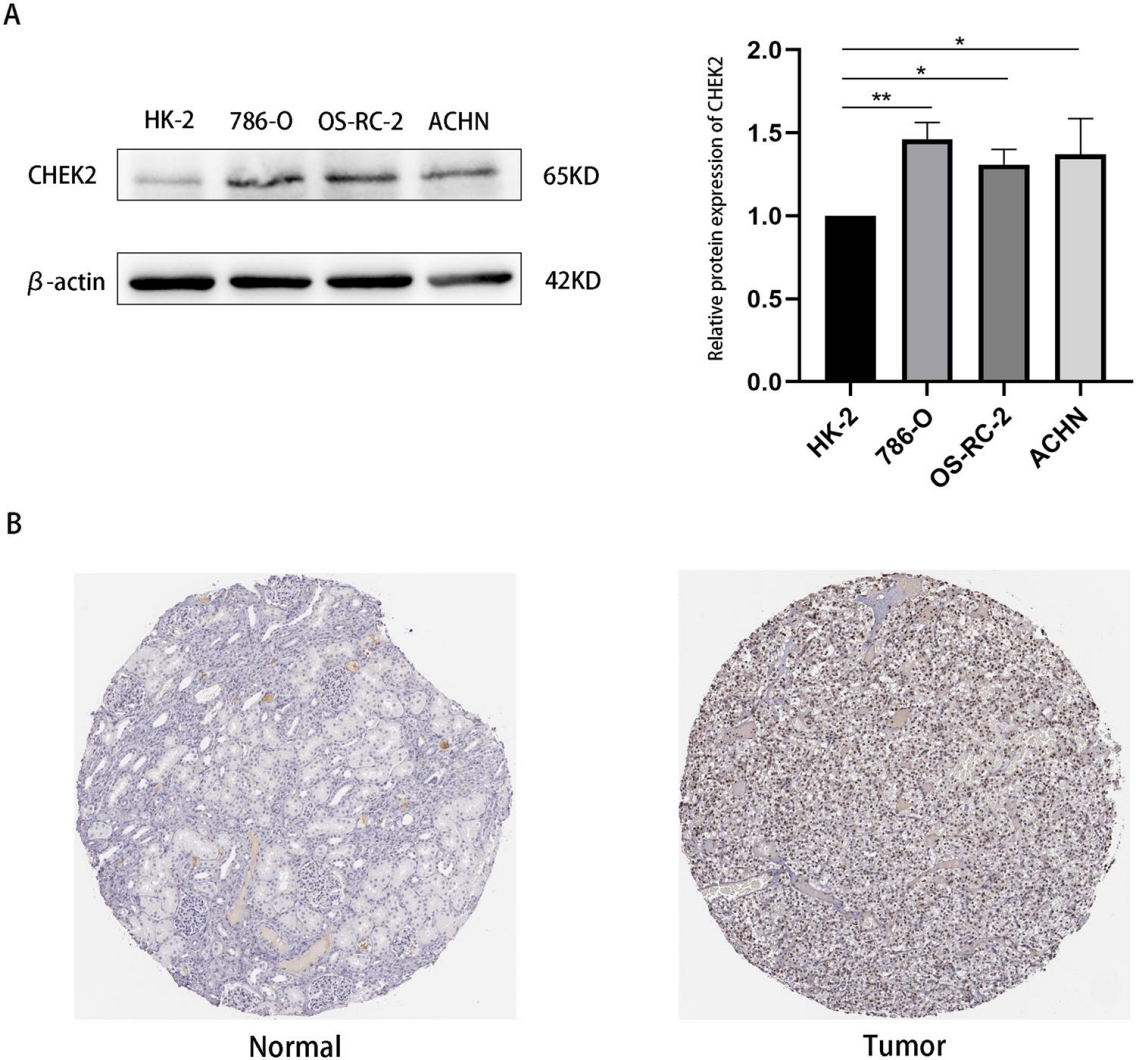
Validation of CHEK2 expression in clinical samples and cell lines. (**A**) Western blot analysis of the CHEK2 expression in the HK-2, 786-O, OS-RC-2 and ACHN cell lines (n = 3 biological repeats). (**B**) Representative immunohistochemistry images of CHEK2 in ccRCC cancer tissue and corresponding normal tissue. *P < 0.05, **P < 0.01, ***P < 0.001.

To verify the findings obtained from the above databases, we determined CHEK2 protein levels in ccRCC cell lines using the Western blot assay. It was observed that the 786-O, OS-RC-2, and ACHN cell lines showed increased CHEK2 expression compared to HK-2 cells (Fig. 2A). Full-length blots/gels are presented in Figures S1 and S2. Furthermore, immunohistochemistry (IHC) images from the HPA database confirmed that CHEK2 protein expression elevated in ccRCC samples (Fig. 2B). Thus, the findings of experiments on clinical samples and cell lines corroborated the findings of bioinformatics analysis.

### CHEK2 expression is tightly associated with clinicopathological features

**Figure 3:**
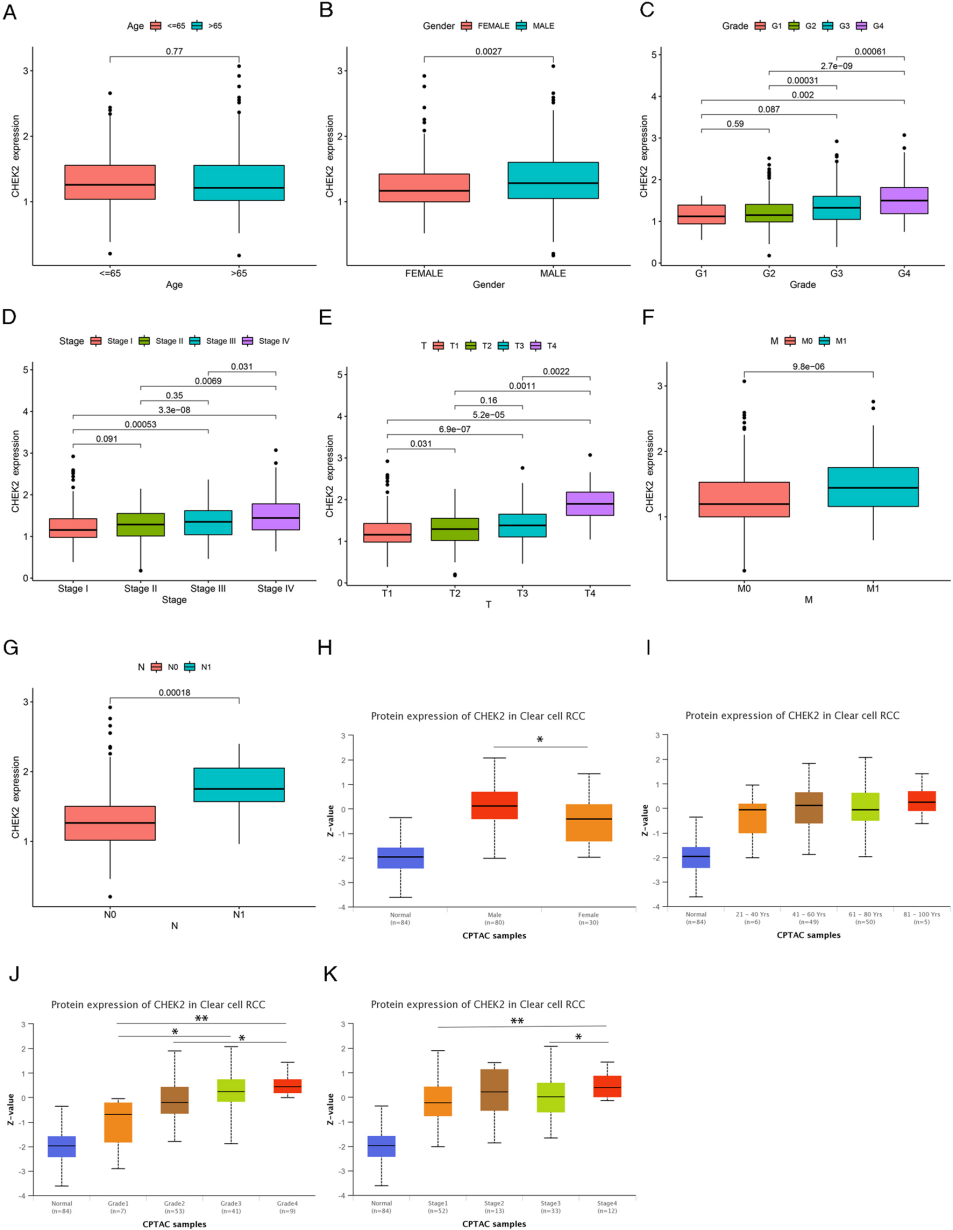
Correlations between the expression of CHEK2 and clinicopathological features in ccRCC. A boxplot depicted the mRNA expression of CHEK2 was no significant correlation with age (**A**). The mRNA expression of CHEK2 was significantly higher in male (**B**), high-grade (**C**), high pathological stage (**D**), high T stage (**E**), distant metastases (**F**), and lymph node metastasis (**G**). Boxplots illustrated the relationships between CHEK2 protein expression levels and gender (**H**), age (**I**), grade (**J**), stage (**K**). *P < 0.05, **P < 0.01, ***P < 0.001.

The CHEK2 mRNA expression was evaluated in various clinical categories to understand the effect of CHEK2 on ccRCC using data from the TCGA-KIRC cohort. According to the results, except for age (Fig. 3A), elevated CHEK2 mRNA levels were closely related to gender (Fig. 3B), grade (Fig. 3C), clinical stage (Fig. 3D), and T/N/M stages (Fig. 3E–G). Thus, CHEK2 could be a potential biomarker for high-risk ccRCC patients. Furthermore, this study investigated the protein expression of CHEK2 in CPTAC ccRCC samples from the UALCAN database. According to the findings, CHEK2 protein expression was shown to be higher in male, high-stage, and high-grade ccRCC patients, independent of age (Fig. 3H–K). This outcome agreed with the findings from the TCGA database.

### Increased CHEK2 expression is linked to unfavorable prognosis in ccRCC patients

**Figure 4:**
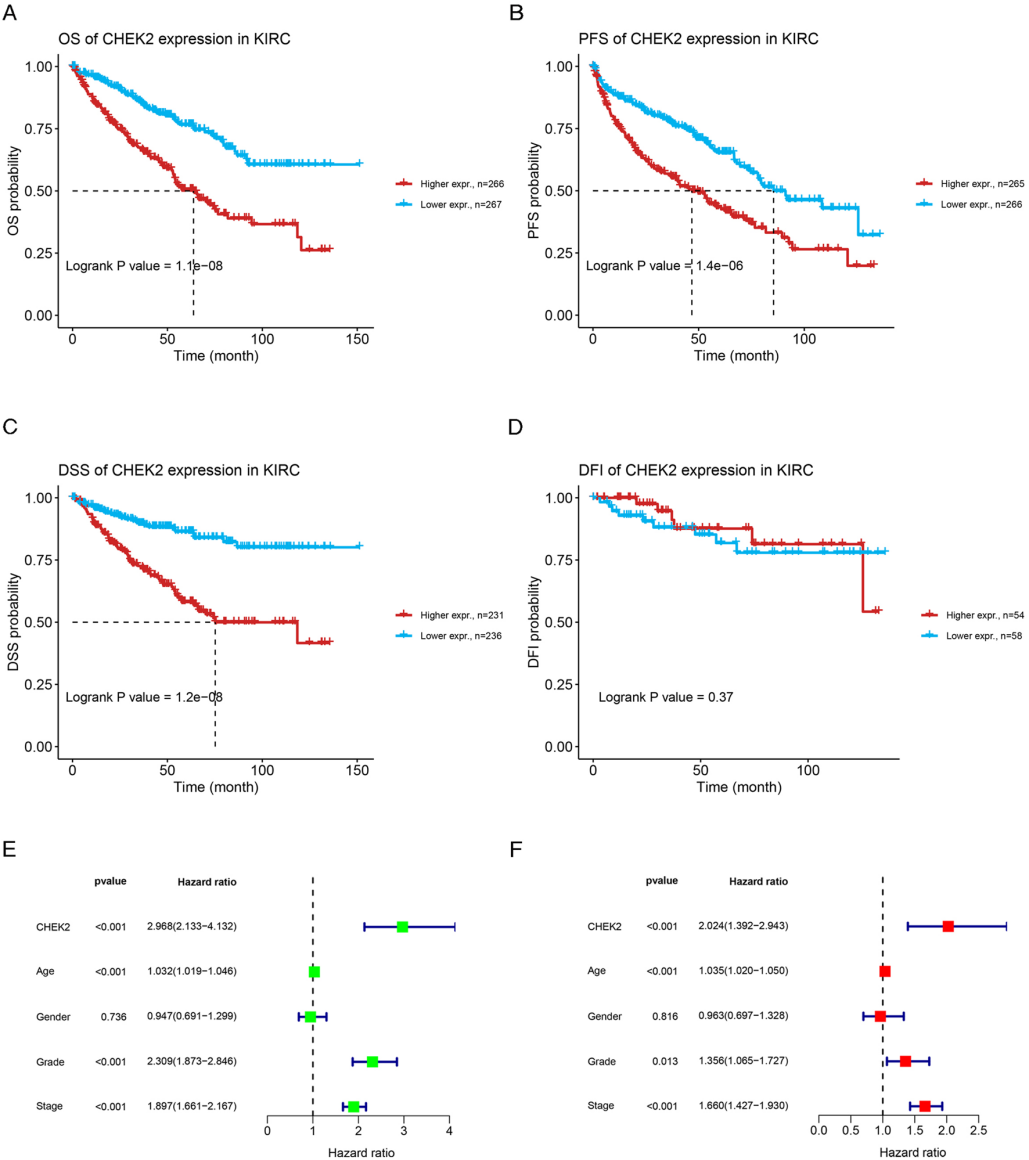
Assessments of CHEK2 expression on survival of ccRCC patients. (**A**) Overall survival, (**B**) Progression-free survival, (**C**) Disease- specific survival, and (**D**) Disease-free survival in ccRCC patients between high- and low-CHEK2 expression groups. Univariate Cox regression analysis (**E**) and multivariate Cox regression analysis (**F**) of CHEK2 and clinical characteristics on prognosis of ccRCC patients.

Through the GSCA website, the significance of CHEK2 expression in predicting the prognosis of ccRCC patients was assessed. According to the Kaplan–Meier survival analysis, the OS of the high-CHEK2-expression group decreased in relative to the low-CHEK2-expression group (Fig. 4A). Besides, CHEK2 expression was adversely linked with PFS (Fig. 4B) and DSS (Fig. 4C) in ccRCC patients. Nevertheless, the CHEK2 level was not remarkably related to DFI (Fig. 4D) in ccRCC patients. Then, we assessed the independent prognostic factors using Cox regression. Both univariate (Fig. 4E) and multivariate Cox regression (Fig. 4F) analyses indicated that CHEK2 expression, age, grade, and stage were independent predictive variables for the prognosis of ccRCC patients.

### Functional enrichment of DEGs in low- versus high-CHEK2-expression groups

**Figure 5:**
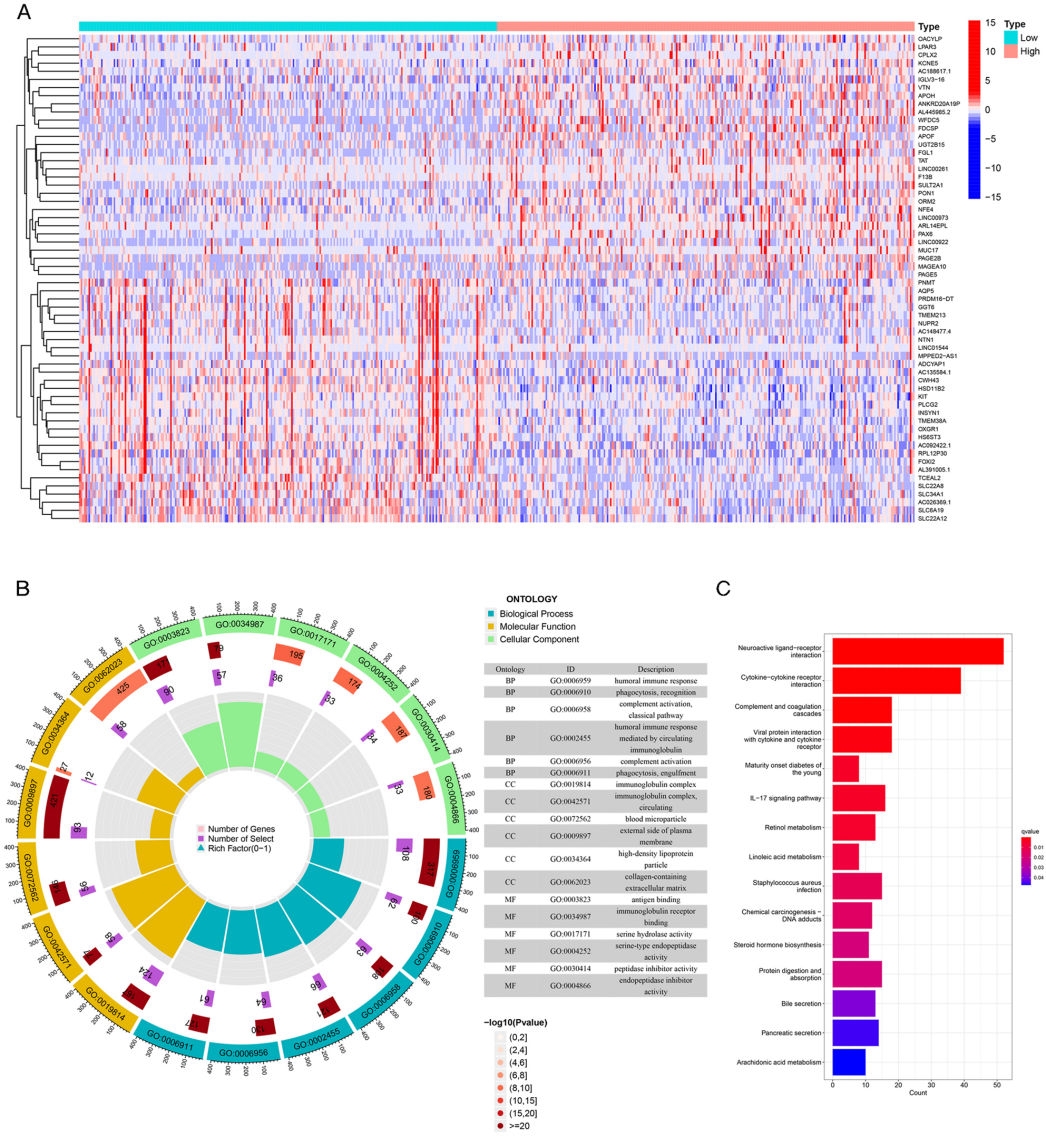
GO and KEGG enrichment analyses of DEGs between low and high CHEK2 expression groups. (**A**) Heatmap of the top 60 DEGs of the two groups. (**B**) GO enrichment circle diagram of DEGs. (**C**) Bar plot of 15 most relative KEGG pathway enriched terms.

Results of the differentiation analysis revealed that the top 60 DEGs included OACYLP, LPAR3, CPLX2, and KCNE5 (Fig. 5A). GO and KEGG analyses were conducted to explore the biological functions and pathways enriched by DEGs in low- and high-CHEK2-expression groups. GO terms were associated with immune-related BPs, including phagocytosis, humoral immune response, and complement activation (Fig. 5B). Moreover, the KEGG analysis proved that the DEGs were correlated with complement and coagulation cascades, cytokine-cytokine receptor interaction, IL-17 signaling pathway, linoleic acid metabolism, arachidonic acid metabolism, and chemical carcinogenesis-DNA adducts (Fig. 5C). These results indicated that immune-related pathways, lipid metabolism pathways, and certain compounds made an impact on the contribution of CHEK2 to the development of ccRCC.

### CHEK2 expression is correlated with immune-cell infiltration and TMB

**Figure 6:**
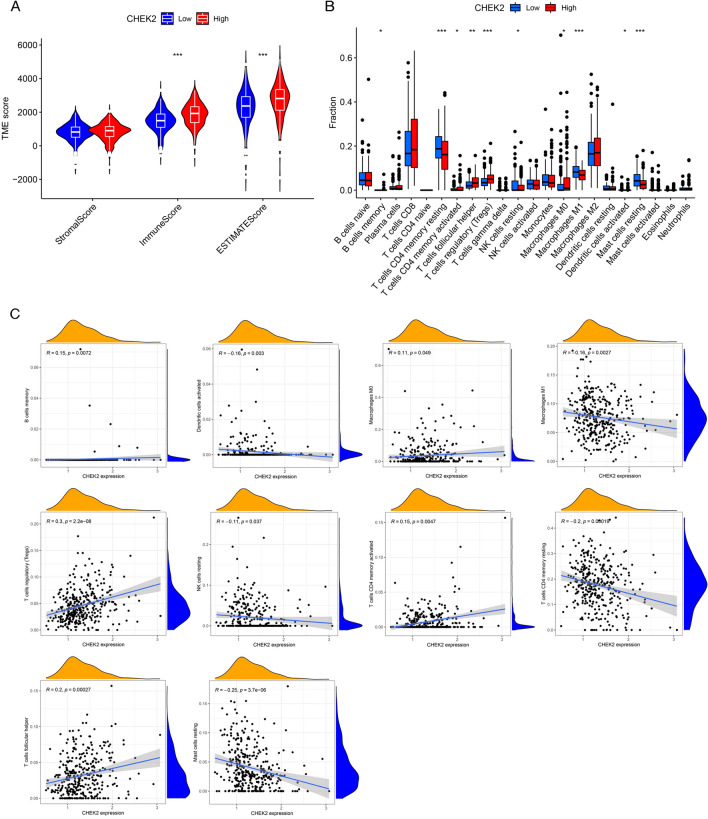
Immune landscape of patients in the low and high CHEK2 expression groups. (**A**) TME score. (**B**) The proportion of 22-type immune cells in the two groups. (**C**) Correlations between expression level of CHEK2 and the infiltration levels of different lymphocyte types. *P < 0.05, **P < 0.01, ***P < 0.001.

**Figure 7:**
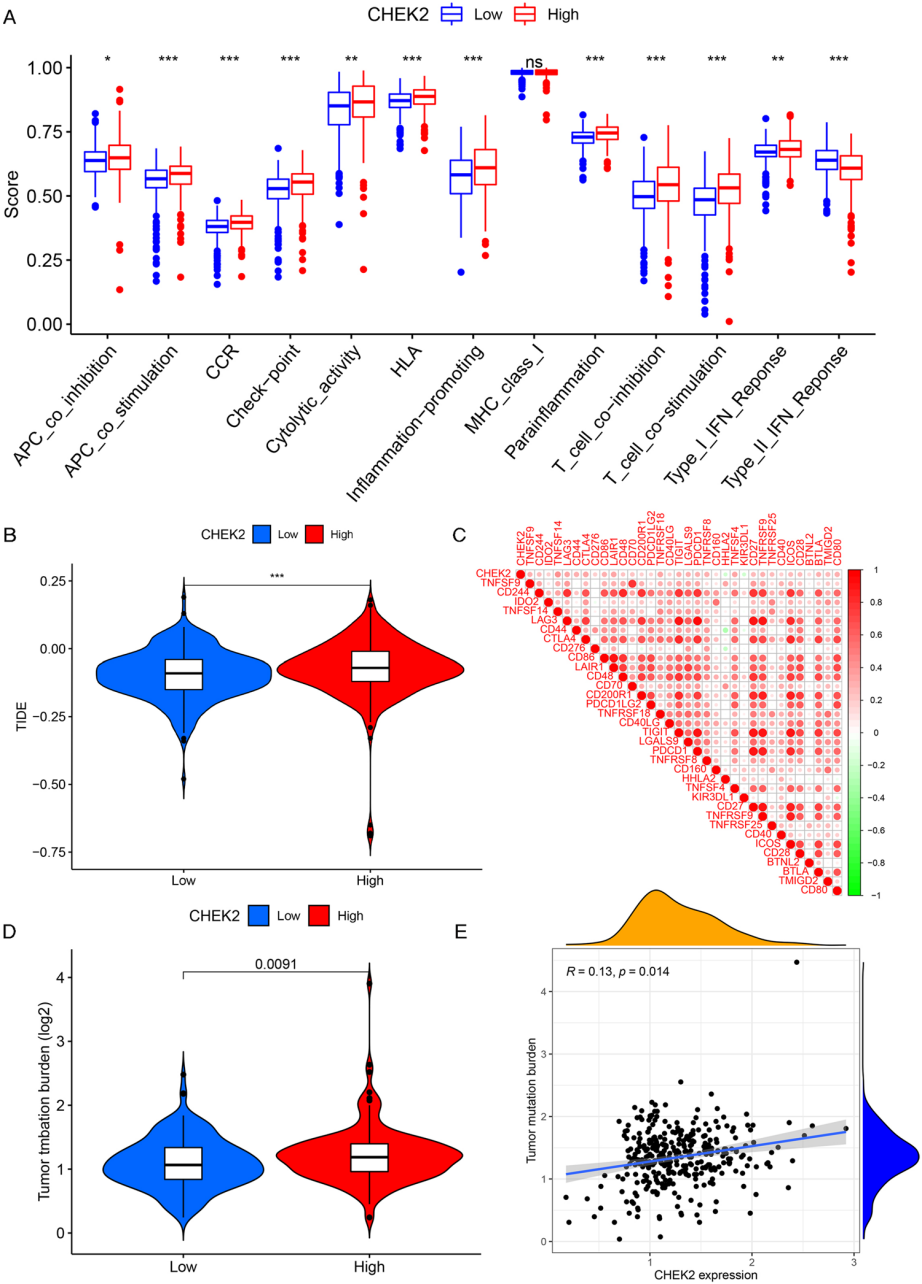
The correlations between immune-related function, immune checkpoints, immunotherapy response, TMB, and CHEK2 expression. (**A**) Immune-related function between high and low expression groups. (**B**) TIDE score. (**C**) The correlation between CHEK2 expression and immune checkpoints. (**D**) TMB differences in the two groups. (**D**) Positive correlation between TMB and CHEK2 expression. *P < 0.05, **P < 0.01, ***P < 0.001.

On the basis of the findings of enrichment analysis, the correlation between the expression level of CHEK2 and immune-cell infiltration was explored. The outcome of the ESTIMATE analysis revealed higher immune/ESTIMATE scores in patients who had high expression of CHEK2 (Fig. 6A). The CIBERSORT analysis indicated that the high-expression group had markedly increased infiltrating proportions of memory B cells, follicular helper T cells, CD4 memory-activated T cells, regulatory T cells (Tregs), M0 macrophages, and activated dendritic cells compared to the low-expression group. On the other hand, the high-expression group showed markedly decreased proportions of CD4 memory resting T cells, resting NK cells, M1 macrophages, and resting mast cells compared to the low-expression group (Fig. 6B). Then, we evaluated the effect of CHEK2 on specific lymphocyte subtypes and observed that CHEK2 expression was positively related to Tregs, follicular helper T cells, CD4 memory-activated T cells, memory B cells, and M0 macrophages but negatively correlated with resting mast cells, CD4 memory resting T cells, M1 macrophages, activated dendritic cells, and resting NK cells (Fig. 6C). We then examined the immune functions between the two groups using the ssGSEA algorithm and observed that the levels of antigen-presenting cells (APCs), cytokine receptor interaction (CCR), checkpoint, cytolysis, human leukocyte antigens (HLA), promotion of inflammation, para-inflammation, T cell functions, and IFN response were significantly different between the groups (Fig. 7A). TIDE analysis revealed low TIDE scores in the low-expression group, indicating a superior immunotherapeutic response (Fig. 7B). Moreover, CHEK2 expression showed positive correlation with most immune checkpoints but negatively correlated with HHLA2 and KIR3DL1 (Fig. 7C). TMB is closely associated with the response to immunotherapy. Consequently, differences in TMB were examined, and it was observed that the TMB in the high-expression group was notably higher than the low-expression counterpart (Fig. 7D). It could be discovered that the expression of CHEK2 was significantly positively related to TMB (Fig. 7E). These results demonstrated that CHEK2 showed relationship to the immune infiltration landscape and the immunotherapeutic response in ccRCC cases.

### Protein interaction network and enrichment analysis of CHEK2

**Figure 8:**
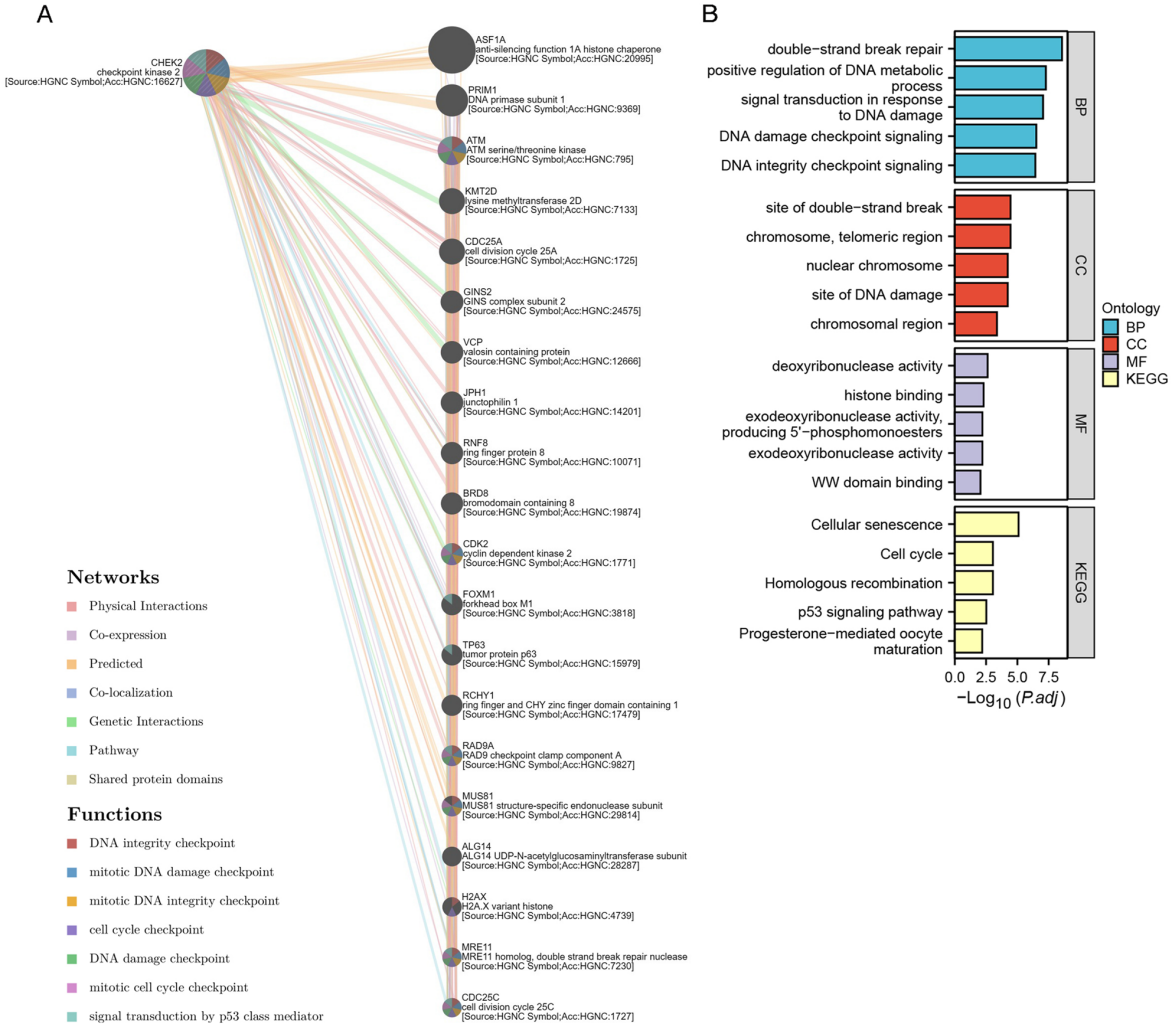
Network and enrichment analysis of the binding proteins for CHEK2. (**A**) CHEK2 associated gene–gene interaction network. (**B**) GO and KEGG analyses.

The top 20 genes associated with CHEK2 were obtained from the GeneMANIA database to conduct GO and KEGG analyses for a better understanding of the effect of CHEK2 expression on ccRCC (Fig. 8A). The primary BPs influenced by CHEK2 expression included signal transduction in response to DNA damage, DSB repair, and DNA damage checkpoint signaling. On the other hand, cellular component (CC) terms were mostly associated with the site of DSB, nuclear chromosome, and the site of DNA damage. Molecular function (MF) terms were mostly associated with deoxyribonuclease activity, histone binding, and exodeoxyribonuclease activity. KEGG pathway enrichment was mainly related to cellular senescence, cell cycle, homologous recombination, and p53 signaling pathway (Fig. 8B).

### Drug sensitivity analysis of CHEK2

**Figure 9:**
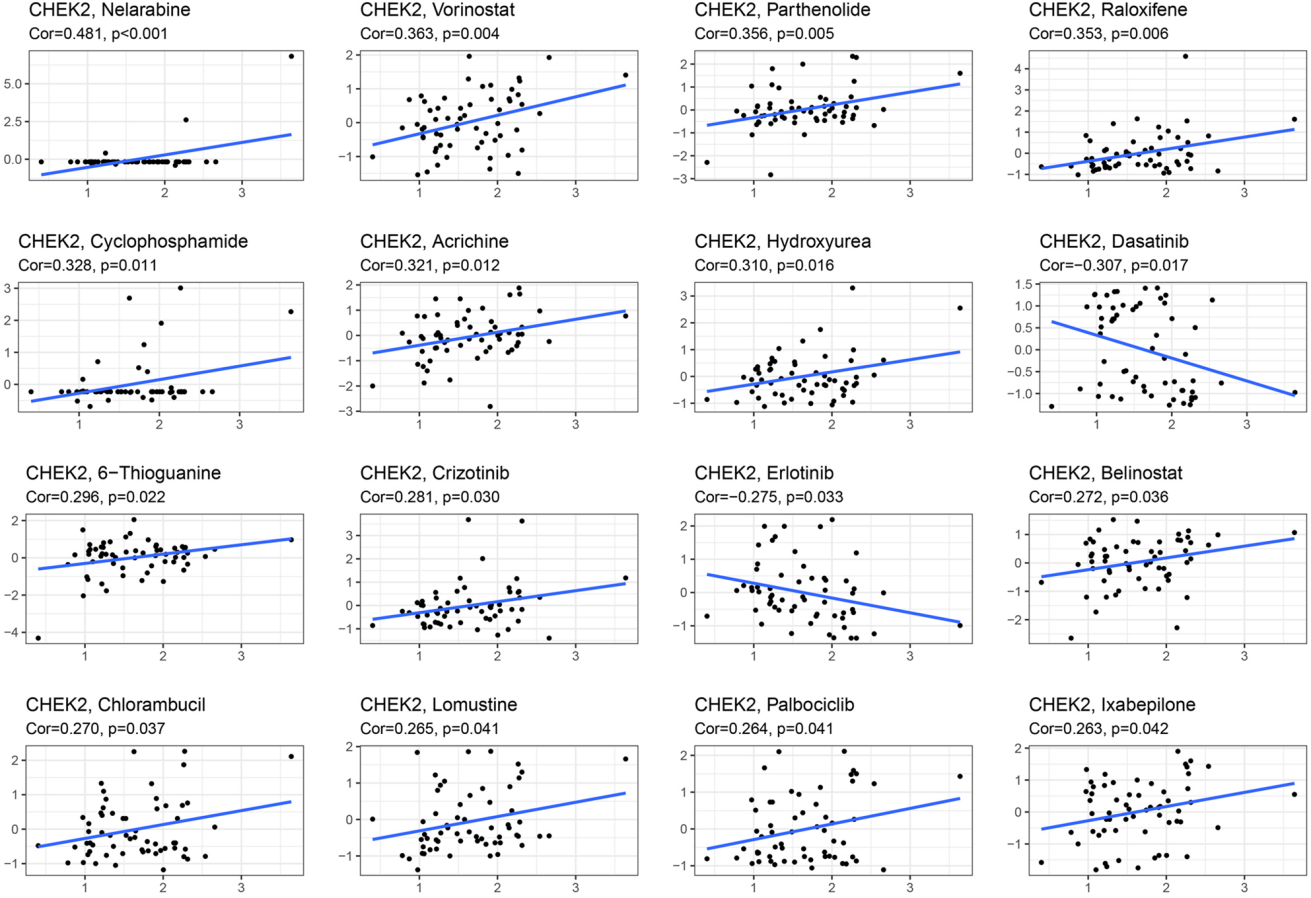
An illustration of the connection between CHEK2 expression and anticipated drug response.

The CellMiner database was used to further examine the potential association between drug sensitivity and CHEK2 expression. Notably, the expression level of CHEK2 showed positive correlation with drug response among cases undergoing treatment with nelarabine, vorinostat, parthenolide, raloxifene, cyclophosphamide, acrichine, hydroxyurea, 6-thioguanine, crizotinib, belinostat, chlorambucil, lomustine, palbociclib, and lxabepilone (Fig. 9). Also, the expression level of CHEK2 was negatively related to the drugs dasatinib and erlotinib (Fig. 9). These findings suggested that CHEK2 might be linked to resistance against certain targeted drugs, such as dasatinib and erlotinib, which are commonly used in the clinic.

## Discussion

RCC is among the most common urological cancers, and its incidence has continued to elevate during the past few years^20^. ccRCC accounts for the highest RCC cases^21^. Up to a third of individuals with ccRCC present with or acquire metastases, even though the disease can be successfully treated with surgical or ablative techniques if detected early^22^. Although methods for the diagnosis and treatment of ccRCC have considerably advanced over the past few decades, metastatic ccRCC is virtually uniformly lethal^23^. Therefore, the identification of new biomarkers for the early screening, diagnosis, as well as treatment of RCC is critical.

CHEK2 is crucial in signaling DNA damage when DSB occurs. The expression of CHEK2 is constant during the cell cycle. During normal growth, CHK2 occurs in the nucleus of cells as an inactive monomer. After DNA damage, the ataxia telangiectasia mutated (ATM) protein which belongs to the phosphatidylinositol 3-kinase (PI3K) family, phosphorylates CHK2 and causes a conformational shift that triggers dimerization and autophosphorylation followed by the dissociation of CHK2 into active monomers. These activated CHK2 phosphorylated proteins have a certain effect on DNA repair, cell cycle regulation, and apoptosis^24^. Herein, a protein interaction network was constructed to identify molecules strongly associated with CHEK2, such as ATM, CDC25A, CDC25C, CDK2, TP63, and H2AX. It was observed that most molecules were connected to DNA damage repair, cell cycle, and apoptosis. In addition, this result was also verified by enrichment analysis.

Previous studies suggested that CHEK2 is the tumor suppressor that exerts its effect by postponing the progression of the cell cycle, thus facilitating DNA repair. Alternatively, it may induce cell death to eliminate genetically unstable cells^25,26^. Several subsequent reports in the literature have implicated the role of CHEK2 in susceptibility to cancer^27,28^. However, recent studies have revealed the role of CHEK2 in newer areas. CHEK2 regulates mitochondrial metabolism and is highly expressed in hepatocellular carcinoma patients^29^. It also phosphorylates the FOXK protein to promote autophagy via transcriptional control, which may cause chemoresistance in cancer^30^. Therefore, additional investigations are required to explore the BPs and potential mechanisms underlying the action of CHEK2 in the occurrence and progression of cancer.

In this study, we examined CHEK2 expression levels in various cancers. We observed that almost all tumors, including ccRCC, showed elevated CHEK2 expression compared to non-carcinoma samples. Results of Western blot and immunohistochemistry also confirmed this finding. Moreover, high expression of CHEK2 was related to adverse clinicopathological variables, including pathological grade, clinical stage, and T/N/M stages in ccRCC. Kaplan–Meier survival analysis demonstrated that increased CHEK2 expression predicted poorer survival outcomes in terms of OS, PFS, or DSS. Cox regression analysis suggested that CHEK2 independently predicted the risk of ccRCC. These findings suggested that CHEK2 might become a possible prognostic indicator for ccRCC.

Metabolic reprogramming is a hallmark of ccRCC, encompassing various processes, such as aerobic glycolysis, fatty acid metabolism, as well as the utilization of tryptophan, glutamine, and arginine^31^. In the contemporary era, the integrated application of multi-omics approaches has significantly advanced our comprehension of ccRCC as a metabolic disease^32^. An analysis that integrated metabolomic and transcriptomic data revealed that in ccRCC, shifts in the pattern of glucose metabolism lead to mitochondrial damage, thus promoting cancer development^33^. Likewise, a recent investigation suggested that CHEK2 governs energy production in cancer cells by affecting both glycolysis and mitochondrial functions^29^. Moreover, several systematic analyses of metabolites have elucidated the crucial involvement of aberrant lipid metabolism in ccRCC^34,35^. It highlights the potential efficacy of targeting lipid metabolism pathways as a promising therapeutic strategy for addressing ccRCC. Examining ccRCC transcriptome data, we discovered that the DEGs between high- and low-CHEK2 expression groups were significantly enriched in linolenic acid and arachidonic acid metabolism pathways. Our findings contribute a novel perspective, suggesting that CHEK2 may regulate polyunsaturated fatty acid metabolic pathways in the metabolic reprogramming of ccRCC.

RCC is one of the most immune-infiltrated tumors, wherein immune cells infiltrate the TME and create an ecosystem that influences each aspect of tumor development^36,37^. In this study, DEGs identified in low- versus high-CHEK2-expression groups were subjected to functional annotation which suggested that their functions were mainly related to immune-related BPs, including humoral immune response, phagocytosis, and complement activation. Infiltration of different immune cells in TME heavily affects tumor biological behavior and might also alter responses to systemic therapy^38^. Therefore, we further conducted immune-infiltration analysis and explored the correlation between CHEK2 expression and various immune cells. CHEK2 expression was significantly positively related to Tregs and negatively related to M1 macrophages.

Tregs are widely acknowledged to impede immune surveillance against cancer, suppress anti-tumor immune responses, and promote tumor development. The immunological suppressive mechanisms of Tregs mainly include the suppression of APCs through the CTLA-4 pathway, use of IL-2, secretion of inhibitory factors such as IL-10 and TGF-β, and production of cytotoxic perforin and granzyme^39^. Besides the immunologic processes in response to malignancy, Tregs provide nonimmunologic assistance to cancer by promoting angiogenesis and cancer cell proliferation, as well as mediating epithelial-mesenchymal transition and cancer metastasis^40^. Significantly, an assessment of the immunologic state in four distinct cohorts of RCC patients revealed that patients with high infiltration of Tregs had the worst prognosis in each cohort^41^. Therefore, we hypothesize that the aberrant increase of Tregs in the TME is a major contributor to dismal prognosis in cases with high CHEK2 expression.

One of the primary immune cell types recruited into the RCC microenvironment is the tumor-associated macrophage (TAM), which is usually classified into two subtypes with different functions, classically activated M1 macrophages and alternatively activated M2 macrophages. M1 macrophages generate inflammatory cytokines to perform anti-tumor functions, while M2 macrophages stimulate tumor growth and promote metastasis of the cancer^42^. Results from a recent single-cell RNA sequencing study of ccRCC patients at different clinical stages suggested a consistent decrease in M1 macrophages with the progression of ccRCC from early to locally advanced and metastatic disease^43^. Meanwhile, our findings indicate that the infiltration of M1 macrophages significantly decreased in the group with high CHEK2 expression. Therefore, we assume that CHEK2 expression is associated with the infiltration of M1 macrophages, which could be another mechanism influencing the prognosis of ccRCC.

The management and therapy of metastatic RCC have witnessed significant changes over the past 20 years^44^. Immune checkpoint inhibitors (ICIs) emerged with promising outcomes^45,46^. Although ICIs can provide a long-term benefit in responders, most people still exhibit ICIs resistance, and it is unclear how immune cells and cancer interact causally^47^. Based on findings from several relevant articles, the application of CHEK2 inhibitors in tumors with high CHEK2 expression can sensitize the tumors to ICIs^48,49^. Herein, TIDE scores of all cases in both groups were determined, and it was observed that the group with high CHEK2 expression showed increased TIDE scores, suggesting that this group was less effective for immunotherapy. Therefore, combining CHEK2 inhibitors with ICIs may become an effective therapeutic strategy for treating ccRCC with high CHEK2 expression.

This work has several limitations. First, our data were mainly obtained from public databases. We only examined the CHEK2 protein levels in clinical samples and cell lines. Further biochemical experiments illustrating the potential functions of CHEK2 in ccRCC are required. Second, we mainly concentrated on the correlations between CHEK2, Tregs, and M1 macrophages. Further research on the connection between CHEK2 and other immune cells is required.

## Conclusions

Collectively, the findings of this work indicate that the expression of CHEK2 is upregulated in ccRCC and high CHEK2 expression predicts the dismal prognostic outcome of ccRCC. Additionally, CHEK2 affects tumor immune cell infiltration and the immunotherapeutic response, which possibly predicts a poor prognosis of ccRCC. Our research provides fresh perspectives and insights into a novel potential therapeutic strategy for ccRCC.

### Supplementary Information

Supplementary Figures.

## Data Availability

All relevant data are within the paper.
