# Supplementary material for: CHEK2 is a potential prognostic biomarker associated with immune infiltration in clear cell renal cell carcinoma
Source: Sci Rep. 2023 Dec 11;13:21928. doi: 10.1038/s41598-023-49316-6 (PMC10713979; doi:10.1038/s41598-023-49316-6)
Supplement: Supplementary file 1 — Supplementary Figures. [file 41598_2023_49316_MOESM1_ESM.pdf]

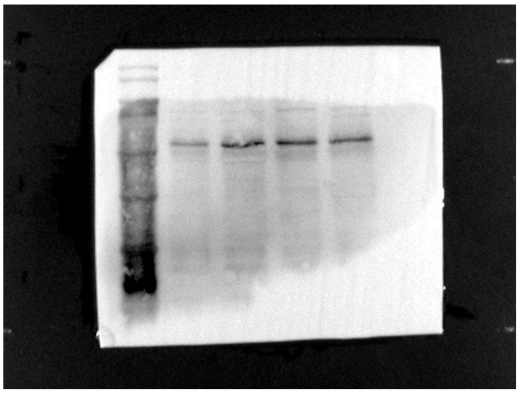

Fig. S1 Full-length blots/gels of CHEK2 from the western blot assay.

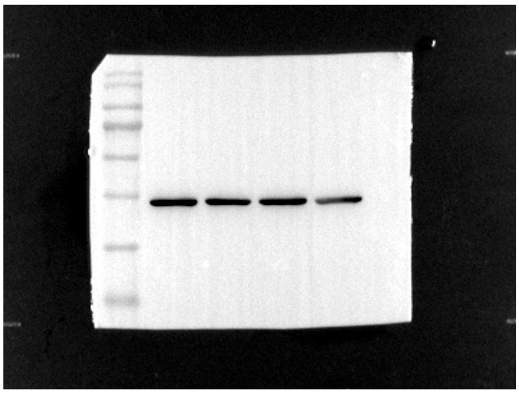

Fig. S2 Full-length blots/gels of β-actin from the western blot assay.
